# Naringenin and Its Derivatives—Health-Promoting Phytobiotic against Resistant Bacteria and Fungi in Humans

**DOI:** 10.3390/antibiotics11111628

**Published:** 2022-11-15

**Authors:** Anna Duda-Madej, Jakub Stecko, Jakub Sobieraj, Natalia Szymańska, Joanna Kozłowska

**Affiliations:** 1 Department of Microbiology, Faculty of Medicine, Wroclaw Medical University, Chałubińskiego 4, 50-368 Wrocław, Poland; 2 Faculty of Medicine, Wroclaw Medical University, Ludwika Pasteura 1, 50-367 Wrocław, Poland; 3 Department of Food Chemistry and Biocatalysis, Faculty of Biotechnology and Food Science, Wrocław University of Environmental and Life Sciences, C.K. Norwida 25, 50-375 Wrocław, Poland

**Keywords:** naringenin, naringenin derivatives, antimicrobial activity, anti-inflammatory activity, antioxidant activity, antifungal activity

## Abstract

Naringenin is a trihydroxyflavanone present in large amount in different citrus fruits, e.g., oranges, pomelos, grapefruits, but also in tomatoes, fenugreek and coffee. It has a wide range of pharmacological and biological effects beneficial to human health. Its antioxidant, anti-cancer, anti-inflammatory, antifungal and antimicrobial activity is frequently reported in scientific literature. In this review we presented the current state of knowledge on the antimicrobial activity of naringenin and its natural and synthetic derivatives as a phytobiotic against resistant Gram-positive and Gram-negative bacteria as well as fungi in humans. Most of the data reported here have been obtained from in vitro or in vivo studies. Over the past few years, due to the overuse of antibiotics, the occurrence of bacteria resistant to all available antibiotics has been growing. Therefore, the main focus here is on antibiotic resistant strains, which are a significant, worldwide problem in the treatment of infectious diseases. The situation is so alarming that the WHO has listed microbial resistance to drugs on the list of the 10 most important health problems facing humanity. In addition, based on scientific reports from recent years, we described the potential molecular mechanism of action of these bioflavonoids against pathogenic strains of microorganisms. As plant-derived substances have been pushed out of use with the beginning of the antibiotic era, we hope that this review will contribute to their return as alternative methods of preventing and treating infections in the epoch of drug resistance.

## 1. Introduction

**Figure 1:**
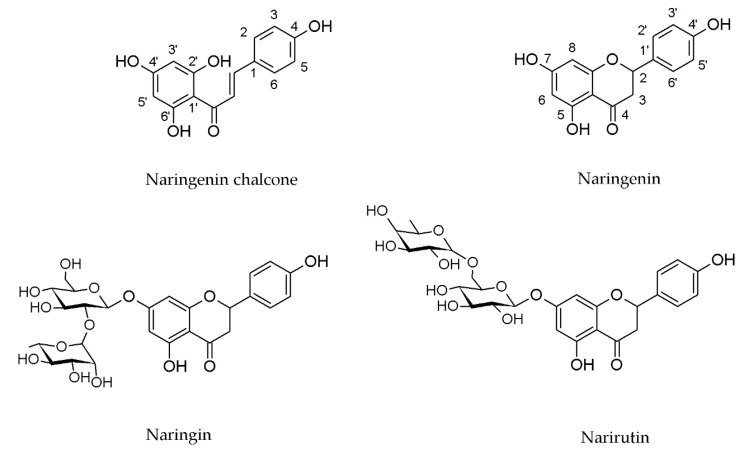
Structures of naringenin chalcone, naringenin, naringin and narirutin.

Naringenin (5,7,4′-trihydroxyflavanone) is an aglycone, which is a derivative of hydrogenated flavone, belonging to the group of flavonoid compounds, which are a part of a huge group of polyphenols. It is also produced by the cyclization of 2′,4′,6′,4-tetrahydroxychalcone (naringenin chalcone) (Figure 1). This bioflavanone occurs naturally in an inactive form as naringin and is converted into its active form, naringenin, by bacteria belonging to the gut microbiome [1]. This, one of the most important representatives of flavonoid compounds, is a component of the everyday human diet, where it is responsible for the color and bitter-sour taste of food. The best sources of it are grapefruit, sour orange, tart cherries, tomatoes, grapes and Greek oregano. It is also found in smaller amounts in bergamot, beans, fenugreek, milk thistle, tea, coffee, cocoa and red wine [2]. Although almost all citrus fruits are source of flavonoids for humans, their concentration varies and depends on the type, variety, harvest time and environmental conditions in which they grow.

Naringenin has a wide range of positive effects on human health. It promotes carbohydrate metabolism, increases antioxidant defense, scavenges reactive oxygen species, modulates the activity of the immune system and also has anti-cancer, anti-inflammatory and anti-atherosclerotic effects [3]. It also has the ability to cross the blood-brain barrier, and therefore exerts a variety of positive neuronal effects [4]. These versatile properties of naringenin can be helpful in the prevention and combating of such disorders as obesity, hyperlipidemia, hypertension, atherosclerosis, diabetes and even Alzheimer’s disease.

The last decade has enriched world literature with reviews about naringenin and its natural derivatives in terms of their biological properties, e.g., anti-cancer [5], antioxidant, anti-hyperlipidemic, anti-obesity, hepatoprotective [6,7], anti-inflammatory, anti-diabetic and anti-neurodegenerative activities [8,9]. This paper summarizes the current knowledge on the antimicrobial activity of this bioflavonoid and its natural but also synthetic derivatives. The analysis considers clinical strains of both bacteria—Gram-positive and Gram-negative—characterized by a wide panel of resistance, but also non-pathogenic strains. Furthermore, we collected information about antifungal activity of these substances. In addition, we presented detailed information explaining the mechanism of action of these compounds based on the world literature of recent years.

## 2. Content of Naringenin and its Glycosides in Plants and Products of Plant Origin

Naringenin is widespread in various citrus fruits (grapefruits, lemons, limes, oranges, mandarins, pomelos and bergamots), but also in vegetables, herbs and products of plant origin such as juices or wine. The content in plants is different and depends on the variety and part (flavedo, albedo, pith, seeds, membranes) of fruits, and the diversity of the composition in products of plant origin results from the way of preparation of the final product [10,11]. In plants, naringenin usually occurs as glycoside form: naringin (naringenin-7-rhamnoglucoside) and narirutin (naringenin-7-glucoside) (Figure 1).

Grapefruit (*Citrus paradisi*) is a fruit known for its abundant content of naringenin and its derivatives. The comparison of the content of flavonoids in different varieties of grapefruit proved that naringin is a dominant compound. Its content in white grapefruit was 16.90 mg/100 mg, and in pink/red grapefruit 13.87 mg/100 mg. Moreover, the second flavonoid occurring in the greatest amount was narirutin, with an amount of 5.36 mg/100mg and 3.34 mg/100 mg in white and pink/red variety, respectively [12]. McIntosh et al. also determined the content of naringin in three varieties (Duncan, Marsh and Thompson) of grapefruit in different parts of the fruit. Their research, conducted on different plant tissues, showed the highest amount of naringin in the back membrane, which is near albedo (17,994–27,330 ppm) in all tested varieties. The lowest content of this glucoside form of naringenin has been found in juice vesicles (295–377 ppm) [10]. Additionally, Victor et al. searched for the best extraction method of naringin from grapefruit peel. As a result of their research, scientists obtained the highest amount of naringenin glycoside from wet albedo using hot methanol (0.7375 g of naringin/69.84 g fresh albedo) [11]. Among ready-to-eat products of plant origin characterized by a high content of naringenin and its derivatives, grapefruit juices are distinguished. Ho and co-workers determined the content of naringenin and naringin in grapefruit juices obtained from four different varieties of grapefruits by squeezing the fruits by hand or by squeezer. In more than half the results, the amount of naringin in juices obtained by squeezer was two times higher than by hand (141–656 mg/L grapefruit juice). Furthermore, the concentration of aglycon–naringenin in both types of squeezing was definitely lower, in range of 2.2–80 mg/L grapefruit juice [13].

Naringin is also observed in pomelo fruits (*Citrus grandis*). Sudto et al. performed different types of extraction on pomelo peels, which confirmed the content of naringenin-7-rhamnoglucoside up to 2.4% [14]. Moreover, Lin and co-workers tested three different cultivars of pomelo, and each of them were fragmented to exocarp, mesocarp, lamella, WBW (waste blanching water) and WBE (waste blanching water from exocarps). The naringin concentration was highest in the WBW part of fruit in every variation and amounted to 25.53 mg/g [15].

Another citrus fruit especially abundant in naringin is sweet orange (*Citrus sinensis*). High performance liquid chromatography (HPLC) is an effective and frequently used method to evaluate the content of compounds. Ni et al. using this method determined the amount of flavonoids in citrus juice from *Citrus sinensis*. Authors reported the naringin was present in a concentration of 22.06 ± 0.50 µg/mL. However, naringenin was not detected in the tested concentration range [16]. It is also interesting that in citrus juices tested by Silva et al., naringenin and naringin were present in low concentrations of 0.11–0.176 mg/100 mg and 0.011–0.030 mg/100 mg, respectively [17]. Yalim and co-workers also evaluated the naringin content of sweet orange juices obtained from three different varieties of oranges from four various regions. One type of juice was received by squeezing fruits by hand and second one by blending the orange peels (albedo and flavedo) and mixed with deionized water. The concentration of naringin in orange peel juice was about 2–20 times higher than in orange juice [18].

A scientific group from Spain determined the content of flavonoids in citrus juice obtained from four *Citrus* species: *C. paradisi*, *C. aurantium*, *C. reticulata* and *C. sinensis*. The concentration of naringin and naringenin were 338.36 mg/100 g and 2.35 mg/100 g, respectively [19]. Also, Dhuique-Mayer et al. evaluated the amount of naringenin glycoside from citrus juices from eight varieties of sweet oranges and also from clementine (*Citrus clementina*) and mandarin (*Citrus deliciosa*). The concentration of narirutin in tested juices was in the range of 37.2–98.4 mg/L [20].

Besides citrus fruits, the presence of naringenin was observed in *Musa paradisiaca* L. Behiry et al. determined the concentration of naringenin in banana peel methanolic extract at the level of 8.47 mg/100 g dry extract [21]. Naringenin and its derivatives were also detected at a much lower concentration by Dębski et al. in seven-day-old sprouts of fenugreek (*Trigonella foenum-graecum* L.). Scientists specified the concentration of naringenin and esters of naringenin at the level of 1.4 µg/100 g DW (dry weight). However, glycosides of naringenin were observed in a concentration of 6.3 µg/100 g DW [22]. In the case of green beans of coffee, Alkaltham et al. reported the concentration of naringenin at the level of 1.7 mg/100 g. Furthermore, beans roasted in a microwave or in a conventional oven contained a three-fold higher amount of naringenin with concentrations of 6.04 mg/100 g and 6.05 mg/100 g, respectively [23].

Naringenin also occurs in vegetables, e.g., in tomatoes. Paganga and co-workers determined the concentration of naringenin in Spanish tomatoes at a level of 282 mg/kg DW [24]. Interesting work was conducted by Bugianesi et al., which determined the blood content of naringenin after consumption of cooked tomato paste by men. Their results showed that the test meal containing 150 mg of cooked tomatoes contained 3.8 mg of naringenin and the content of naringenin in plasma after 2 h was 0.12 µmol/L [25].

## 3. Other Derivatives of Naringenin

**Figure 2:**
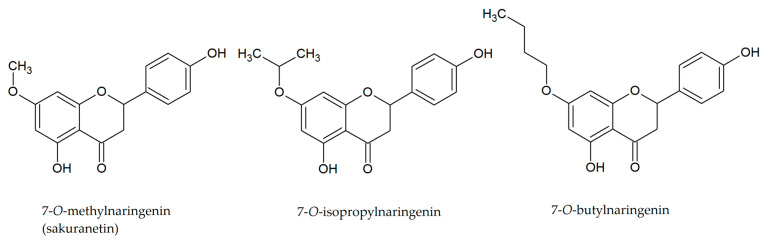
Structures of *O*-alkyl derivatives of naringenin.

Among naringenin derivatives, one of the most described compounds are ether derivatives of naringenin. *O*-alkyl derivatives of naringenin also occur naturally in plants of the Boraginaceae family, e.g., 5-*O*-methylnaringenin, 7,4′-di-*O*-methylnaringenin, 7-*O*-methylnaringenin (sakuranetin) and isosakuranetin (4′-*O*-methylnaringenin) (Figure 2) [26,27,28]. Sakuranetin is a phytoalexin present in rice, which is biosynthesized in plants as an effect of biotic or abiotic stress by naringenin 7-*O*-methyltransferase [29]. There is also a chemical method to obtain 7-*O*-methylnaringenin in a simple reaction of naringenin with methyl iodide or methyl bromide in short time with a high yield [30]. Furthermore, 7-*O*-butylnaringenin is well-known for high activity against *Staphylococcus aureus* MRSA (methicillin-resistant *S. aureus*) [31], but also against *Helicobacter pylori* [32]. The elongation of the alkyl chain attached to naringenin at the C-7 position, and also at the C-4′ position, is a popular reaction in the last decade [30,33,34].

## 4. Properties of Naringenin

### 4.1. Anti-Cancer Activity

The anti-cancer, anti-proliferative and anti-tumor activity of naringenin is primarily related to its ability to repair DNA. Its anti-tumor activity has been demonstrated against breast cancer cells [35], liver cancer [36,37], prostate cancer [38], melanoma [39] and glioblastoma of the brain and spinal cord [40].

Naringenin has been shown to affect both the internal (mitochondrial) and the external (receptor) pathway of apoptosis activation. This bioflavonoid acts in multiple ways at different steps in these pathways. First of all, it blocks the G0/G1 and G2/M phases, and therefore prevents the proliferation of neoplastic cells. It influences the accumulation of the p53 protein, which in turn binds to anti-apoptotic proteins (e.g., Bcl-2), promotes the action of the Bax protein and leads to the formation of pores in the mitochondrial membrane. In this way, it contributes to the loss of the mitochondrial membrane potential, and through this it releases apoptogenic factors, e.g., AIF, cytochrome c [41,42]. It induces apoptosis by damaging the cell nucleus or increasing the ratio of Bax/Bcl-2 cells, i.e., key regulators of apoptosis that control ion flow (K^+^, H^+^, Cl^−^, Ca^2+^) and reactive oxygen species. Thus, it acts as the “death factor” of the neoplastic cell. Under the influence of naringenin, there is also an intracellular accumulation of TGF-β1 (a factor involved in tumorigenesis), and thus reduced secretion and inhibition of the neoplastic process [36].

Recent studies have shown that naringenin, in addition to its effect on apoptosis, also has an inhibitory effect on proliferation (inhibits the phosphorylation of ERK1/2—extracellular signal-regulated kinase 1/2 and JNK—Jun N-terminal kinase) and angiogenesis (inhibits the expression of Tie2—tyrosine-protein kinase receptor-2 and enhances the expression of Ang2—angiopoietin-2) in murine and human melanoma cells (B16F10 and SK-MEL-28 cell lines, respectively) [43].

### 4.2. Anti-Inflammatory Activity

Naringenin inhibits leukocyte recruitment, thus preventing the action of resident macrophages, which produce chemotactic molecules to attract leukocytes to the focus of inflammation (mainly neutrophils). It also acts directly on macrophages by inducing the activation of Nfr2, a factor that initiates the anti-inflammatory response [44]. In addition, naringenin stops the activation of NF-κB, thus contributing to the inhibition of the secretion of pro-inflammatory cytokines, e.g., IL-33, TNFα, IL-1β and IL-6 [8]. It has been shown that this bioflavonoid can suppress the TLR4 receptor, necessary to recognizing bacterial lipopolysaccharide and initiating an inflammatory response in the body [6,8]. Other intracellular proteins (e.g., MyD88—myeloid differentation primary response protein 88; TIRAP/Mal—Tir domain-containing adapter protein; TRIF/TICAM1—TIR domain containing adapter including INF-β; TRAM/TICAM2—Trif-related adapter molecule) are also involved in the recruitment of inflammation, leading to the activation of a cascade of proteins necessary in various conventional pathways (e.g., NO secretion or NF-κB activation). This allows the activated macrophage to participate in inflammation, for example by synthesizing nitric oxide, oxygen free radicals or pro-inflammatory cytokines. However, in the presence of naringenin, it becomes impossible, forasmuch as this bioflavonoid also inhibits nitric oxide synthase (iNOS), preventing the release of NO. It also has an inhibitory effect on the mitogen-activated protein kinase, MAPK, the cascade of which plays an important role in the expression of pro-inflammatory cytokines. In addition, naringenin inhibits the production of superoxide anion and other reactive oxygen species (ROS) while increasing antioxidant capacity [45].

### 4.3. Antioxidant Activity

Naringenin is a powerful antioxidant. It can scavenge free radicals and affect the activity of antioxidant enzymes. Jung et al. reported an increase in the activity of superoxide dismutase (SOD) and catalase (CAT) after the administration of this bioflavonoid in the studied group of people with hypercholesterolemia [46]. On the other hand, in animal studies, the administration of this bioflavonoid decreased lipid peroxidation and increased the level of antioxidants. In addition, it has been shown to effectively neutralize hydroxyl (•OH), superoxide (O^2−^), hydrogen peroxide (H_2_O_2_) radicals, nitric oxide (NO^−^) and DPPH radicals, thereby alleviating liver complications caused by the administration of streptozotocin (STZ) [47]. The same authors, using in vivo studies on mice, showed an increase in the activity of SOD, CAT, glutathione peroxidase (GPx), glutathione S-transferase (GST) and glutathione (GSH), i.e., enzymes that are the body’s defense strategy under unfavorable conditions. Naringenin’s antioxidant abilities are most likely due to its ability to chelate trace amounts of metals, such as iron and copper, which contribute to enhancing ROS production [48]. In addition, naringenin has the ability to donate electrons or a hydrogen atom, leading to the oxidation of superoxide and hydroxyl radicals generated by hydrogen peroxide, therefore causing an enhancement of antigenotoxic activity [49].

### 4.4. Effects on the Nervous System

Naringenin inhibits pain induced by inflammatory stimuli, such as phenyl-β-benzoquinone, acetic acid, formalin, complete Freud’s adjuvant, capsaicin, carrageenan, superoxide anion and LPS [50].

It has been shown that this bioflavonoid has an antinociceptive effect and enhances the pain tolerance of the nervous system in vivo [4]. Inflammatory cells released by pro-hyperalgesic cytokines (such as IL-33, TNF-α, IL-1β and IL-6), activate the nociceptor neurons and induce pain sensitization, leading to pain [51]. The ability of naringenin to inhibit NF-κB activity and induce Nfr2 activation indicates its indirect influence on the activity of nociceptor neuron. It has been shown that naringenin inhibits NF-κB-dependent production of TNF-α and IL-1β, i.e., cytokines that induce nociception by sensitizing neurons through p38 MAPK phosphorylation [50]. Moreover, naringenin regulates TRP channels, expressed by nociceptor neurons such as TRPV1, TRPM3 and TRPM8. In this way, it contributes to the induction of analgesia, i.e., the phenomenon of pain relief [52].

### 4.5. Antidiabetic Activity

In vitro and in vivo studies prove the role of naringenin in the prevention and treatment of insulin resistance and type 2 diabetes. This bioflavonoid can reduce glucose adsorption by the intestinal brush border, and also reduces the level of this sugar in the kidneys. The in vivo research of Li and co-workers on albino rabbits provides evidence that naringenin, by inhibiting intestinal glucose absorption as well as renal reabsorption, directly contributes to the attenuation of hyperglycemia. Furthermore, in vitro studies concerning isolation of the brush border membrane vesicles (BBMV) of the intestines and the renal cortex revealed reduced activity of glucose uptake [53].

Moreover, it has been shown that naringenin also contributes to reabsorption, increased uptake and use of glucose by muscle and adipose tissues. Using primary porcine myotubes confirmed increased glucose uptake by naringenin while reducing intracellular ROS [54,55]. That indicates this bioflavonoid is counteracting insulin resistance. In turn, adipocytes 3T3-L1, treated with naringenin, showed significant reduction of insulin-stimulated glucose levels [56]. Moreover, the signaling cascades, i.e., NF-κB and JNK, inhibited by this aglycone, have suppressed TNF-α induction and adipocyte expression induced by co-culture of macrophages, TLR2. This action directly contributes to the inhibitory involvement in obesity-induced cellulitis [57].

Naringenin has also been shown to have a productive effect on pancreatic β-cells. By “teaching” them, it increases their ability to detect glucose. Bhattacharya et al. proved in their study that β-cells from rat pancreas’, exposed to this bioflavonoid, upregulate insulin-stimulated glucose secretion and, in addition, increase the expression of several genes of these cells: *Glut2; Gck; Ins1,2; Beta2; Act1,2; Pdx1* and *Bcl2*. This suggests that naringenin has a pro-apoptotic effect on pancreatic cells, increasing their sensitivity to glucose [58].

### 4.6. Hepatoprotective Properties

Naringenin reduces triglyceride production and affects gluconeogenesis, contributing to the attenuation of hyperglycemia and hyperlipidemia. The in vivo studies of Ortiz-Andrade et al. confirm this effect. They showed that this bioflavonoid increases the concentration of high-density lipoproteins (HDL) in the serum of Wistar albino rats. In addition, it contributes to the reduction of the activity of enzymes necessary in the process of gluconeogenesis: glucose-6-phosphatase and fructose-1,6-bisphosphatase in the liver [59]. In turn, in vivo studies performed by Sharma et al. confirm that naringin significantly improves the lipid profile in the same animal model on a high-fat diet. Rat serum shows decreased levels of triglycerides, triacylglycerol, LDL cholesterol and non-esterified fatty acids (NEFA), while elevated levels of HDL cholesterol are observed [60].

### 4.7. Antimicrobial Activity of Naringenin

The increasing resistance of bacteria to antimicrobial drugs is a global trend, comparable to an eruption of a sleeping volcano. Currently, for every group of antibiotics, there can be found strains of bacteria which are resistant. This ever-growing problem is due to the intensive use of antibiotics in many areas including food production, veterinary medicine and medicine. Their excessive administration during the COVID-19 pandemic, the treatment of asymptomatic patients and the increasing use of broad-spectrum antibiotics (in the absence of narrow-spectrum ones) promotes this phenomenon and facilitates its propagation [61,62,63]. This overuse leads to the development of multidrug-resistant phenotypes: VRE (vancomycin-resistant *Enterococcus*), MRSA, ESBLs (extended-spectrum beta-lactamases), KPC (*Klebsiella pneumoniae* carbapenemase) and NDM-1 (New Delhi metallo-β-lactamase-1). Naringenin and its derivatives have antimicrobial properties, especially against Gram-positive bacteria, such as *S. aureus*, including antibiotic resistant strain MRSA. At the moment, the results of clinical trials of this bioflavonoid as an antimicrobial agent are poor, and there are no registered clinical trials in the databases of both the U.S. and E.U. However, its pharmacological safety was proven for doses as high as 900 mg (escalated safe total dose) [64], so its usage as an antimicrobial drug can be explored in future.

## 5. Antimicrobial Activity against Gram-Positive Bacteria

Multidrug-resistant (MDR) strains of bacteria are increasingly being isolated mainly in hospital treatment, but also in non-hospital treatment. The prevalence of CA-MRSA (community-associated MRSA) strains is becoming particularly alarming. The Center for Disease Control and Prevention (CDC) listed MRSA as a “serious threat”. This growing resistance brings the need to search for new therapeutic options. Naringenin activity against MRSA is confirmed by number of laboratory investigations and is probably stronger against Gram-positive bacteria than Gram-negative, as shown in a study comparing naringenin activity and mechanism of action against *S. aureus* and *Escherichia coli*. In both species, maximum growth rate was decreasing with increasing concentration of naringenin. However, for *S. aureus,* the inhibition was much more significant, because for a concentration of 1.47 mM, an almost three-fold decrease in viability of this strain was shown. Also, time to reach the stationary phase of growth for *S. aureus* was elongated by naringenin solution. Complete growth inhibition of this strain was observed up to 14 h in the presence of this bioflavonoid at a concentration of 2.20 mM. In the same study, membrane fluidity and fatty acid profiles were investigated, showing that submission to naringenin altered membrane composition in favor of anteiso-branched fatty acids, resulting in increased membrane fluidity. This mechanism of action can suggest that difference in naringenin activity against Gram-positive and Gram-negative bacteria can emerge from differences in their membrane structure [65]. Also, Wang and co-workers, in their other studies, showed that naringenin can interact with the bacterial cell in a variety of ways. This bioflavonoid has an effect on both membrane fatty acids and proteins, and also can bind to the DNA of *S. aureus* [66].

**Scheme 1:**
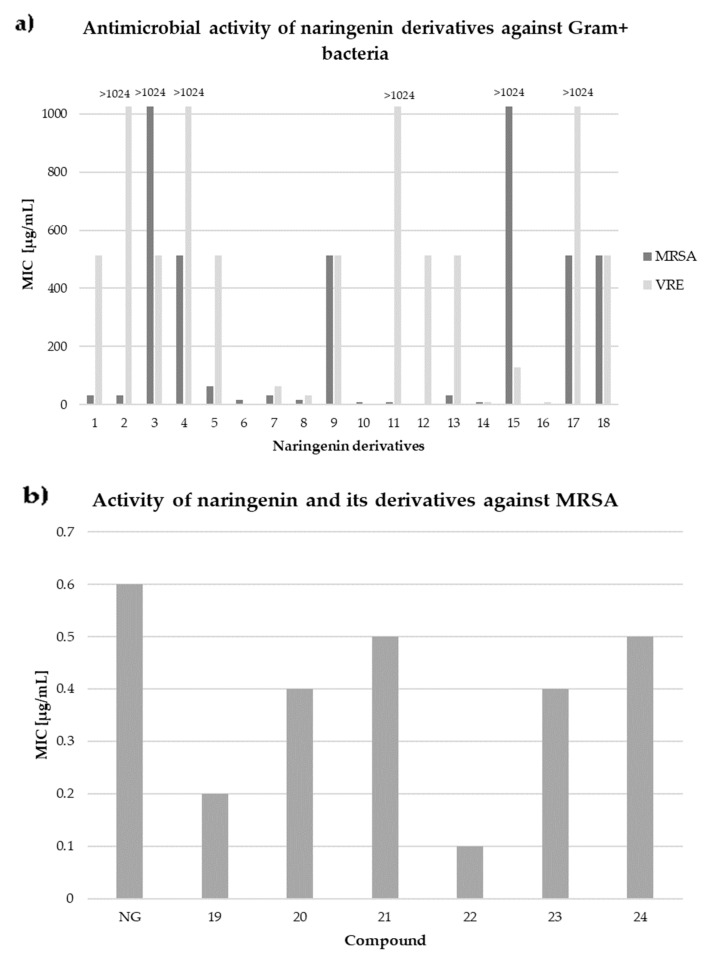
In vitro activity of naringenin and its derivatives against Gram-positive bacteria. (**a**) 1—7-*O*-methylnaringenin; 2—7-*O*-methylnaringenin oxime; 3—7,4′-di-*O*-methylnaringenin; 4—7,4′-di-*O*-methylnaringenin oxime; 5—7-*O*-ethylnaringenin; 6—7-*O*-ethylnaringenin oxime; 7—7-*O*-isopropylnaringenin; 8—7-*O*-isopropylnaringenin oxime; 9—7,4′-di-*O*-isopropylnaringenin; 10—7,4′-di-*O*-isopropylnaringenin oxime; 11—7-*O*-butylnaringenin; 12—7-*O*-butylnaringenin oxime; 13—7-*O*-pentylnaringenin; 14—7-*O*-pentylnaringenin oxime; 15—7-*O*-hexylnaringenin; 16—7-*O*-hexylnaringenin oxime; 17—7,4′-di-*O*-hexylnaringenin; 18—7,4′-di-*O*-hexylnaringenin oxime (based on Duda-Madej et al. [67]). (**b**) NG—naringenin; 19—6-fluoronaringenin; 20—6-chloronaringenin; 21—6-iodonaringenin; 22—6,8-difluoronaringenin; 23—6,8-dichloronaringenin; 24—6,8-diiodonaringenin (based on Mohammed et al. [68]).

A mechanism of action different than that of standard antibiotics can explain why naringenin and its derivatives are active against antibiotic-resistant pathogens such as MRSA. The results of research in this direction are very promising. In a study from 2013, MIC of this bioflavonoid against MRSA (bovine isolate) was measured to be 20 mM, while for its derivative, 7-*O*-butylnaringenin, it was only 0.625 mM [31]. In a more complex study on human isolates, MIC of 12 various naringenin derivatives ranged between 4 and 64 µg/mL. The substances with the greatest potential against MRSA (clinical isolate from blood infections) were: 7-*O*-butylonaringenin oxime and 7-*O*-hexylnaringenin oxime with MIC value of 4 µg/mL; 7-*O*-butylnaringenin, 7-*O*-pentylnaringenin oxime and 7,4′-di-*O*-isopropylnaringenin oxime with MIC value of 8 µg/mL; 7-*O*-ethylnaringenin oxime and 7-*O*-isopropylnaringenin oxime with MIC value of 16 µg/mL. Of these compounds, 7-*O*-isopropylnaringenin oxime showed the lowest MBC value at 32 µg/mL. The same study investigated interactions between the actions of bioflavonoid-based substances and antibiotics against MRSA, showing a synergistic effect between particular naringenin derivatives, such as 7-*O*-isopropylnaringenin oxime and 7-*O*-hexylnaringenin oxime, which lowered MIC for gentamicin eight-fold, MIC for erythromycin (four-fold) and MIC for gentamycin/erythromycin (eight-fold) for 1 and 2 derivative, respectively [67]. In another study, the addition of naringenin and its fluorinated derivatives proved to decrease MIC of ciprofloxacin for MRSA (hospital isolate) by up to 50-fold [68]. Scheme 1a,b show the results of both authors discussed above. However, one study suggested antagonistic interaction between β-lactam antibiotics and naringenin. In this study, bioflavonoid inhibited growth of both MRSA and MSSA (methicillin-sensitive *S. aureus*), but the growth was visible around penicillin and oxacillin discs and methicillin strips [69]. In contrary, another study revealed synergistic interaction between naringenin and oxacillin against MRSA, resulting in lesser cell growth measured as cell density [70].

Since biofilm formation by pathogenic bacteria is considered a major virulence factor (it protects against immune response mechanisms and the targeted action of antimicrobial agents), fighting is a key step in infection control. The useful property of naringenin in this field is its ability to inhibit the formation of biofilm. Such action was proven in the case of *Streptococcus mutans*. In this study, naringenin solution with a concentration of 200 μg/mL almost totally inhibited the formation of biofilm, while 100 μg/mL had about 70% effect and 50 μg/mL was effective at 50%, respectively. Not only growth of bacteria was inhibited, but also their surface hydrophobicity was increased and aggregation reduced. The same study used PCR to determine expression of genes related with biofilm formation: *gtfB, gtfC, comD, comE,* and *luxS*. Those were proven to be suppressed by this bioflavonoid [71]. An analogical study was performed for MRSA and Δ*agr* mutant (mutation inserted in the accessory gene regulator quorum sensing (QS) system), revealing lower expression of *icaAD* gene and thus lower levels of biofilm formation after incubation in naringenin [70].

One more characteristic of naringenin is that it inhibits production of α-toxin, one of the *S. aureus* cytotoxins. This property was confirmed for three different *S. aureus* strains (ATCC 29213, ATCC 10832, and 8325-4) by measurement of haemolytic activity, which decreased to 5.79%, 9.27% and 4.91% compared to the control group. The α-toxin-reduced expression was confirmed using western blot analysis. Interestingly, for one of the tested *S. aureus* strains, USA 300, no α-toxin antigen was detected after treatment with naringenin solution at a dose as low as 2 μg/mL. Zhang and co-workers treated, with naringenin solutions, both lung carcinoma epithelial cells (A549 cell line) and *S. aureus* co-culture system, which simulated pneumonia. They showed that this bioflavonoid had the ability to damage cancer cells, as well as cells changed by the disease process, at levels as high as 50%. They determined the cytotoxic dose for a concentration of 9.22 μg/mL of naringenin by measuring lactate dehydrogenase (LDH) activity. Furthermore, the authors tested with in vivo studies the effect of this bioflavonoid on mice infected with an *S. aureus* strain that caused pneumonia. A dose of 100 mg/kg was administered, which corresponded to 26.04 μg/mL maximum plasma concentration. In histological analysis, the lungs of treated mice had only few focal inflammation areas, while in the control group there were extreme signs of injury [72]. Such effects of naringenin may be due to its effects on the immune response and the levels of secreted cytokines, as evidenced by the research of Yao et al. A study conducted on children with bronchial pneumonia showed that bioflavonoid lowers plasma concentration of pro-inflammatory cytokines such as IL-6, IL-8 and TNF-α and increases the level of anti-inflammatory IL-10, with even greater efficiency than azithromycin [73]. Administration of naringenin turned out to be beneficial for children due to reduction of inflammation, shortening disappearance time of clinical symptoms and reducing complications after therapy. This gives positive insight on naringenin as a potent drug for infections; however, no cultures were done, hence MIC/MBC were not measured. Given these optimistic data, we cannot be sure whether the improvement of the patients is a result of the antimicrobial or anti-inflammatory properties of naringenin.

Naringenin and naringin antimicrobial properties can be used in many ways. One of the investigated areas is usage of naringin in orthopedy, as an element of titanium implant coating. Controlled release of bioflavonoid from a metal-organic framework allowed it to both induce osseointegration and prevent *S. aureus* infection [74]. Another way to use naringin to treat infections caused by Gram-positive bacteria is in a complex with gold nanoparticles stabilized by gum tragacanth. Naringenin encapsulated in a gold nanoparticle showed an MIC of 21.98 μg/mL against *Bacillus subtilis* ATCC 11774. This value is significantly lower (more than three-fold) than for the pure bioflavonoid, for which the MIC was 68.43 μg/mL [75]. In the case of *Micrococcus luteus* ATCC 10240 MIC values were similar for pure bioflavonoid and that complexed with gold nanoparticles (25 µg/mL), but the IC_50_ (the concentration at which 50% growth inhibition was observed) was lower for the complex, with values of 371.66 μg/mL and 253.93 μg/mL, respectively. Therefore, flavonoids such as naringenin or naringin and their derivatives can be used in fighting against infectious diseases not only by direct action but also using carriers such as the described gold nanoparticles, or as a component of biomaterials.

In conclusion, naringenin and its derivatives have the potential to be used against Gram-positive bacteria due to number of different properties. They inhibit not only the growth of pathogenic bacteria, including those resistant to classic antibiotics (MRSA), but also reduce biofilm formation and toxin production. Therapeutic use must still be tested in clinical trials, but taking into consideration all these properties, its pharmacological safety and possible synergistic interaction with antibiotics, naringenin and its derivatives have considerable potential to be used in therapy, especially in treatment of infections caused by antibiotic-resistant pathogens such as MRSA.

## 6. Antimicrobial Activity against Gram-Negative Bacteria

**Scheme 2:**
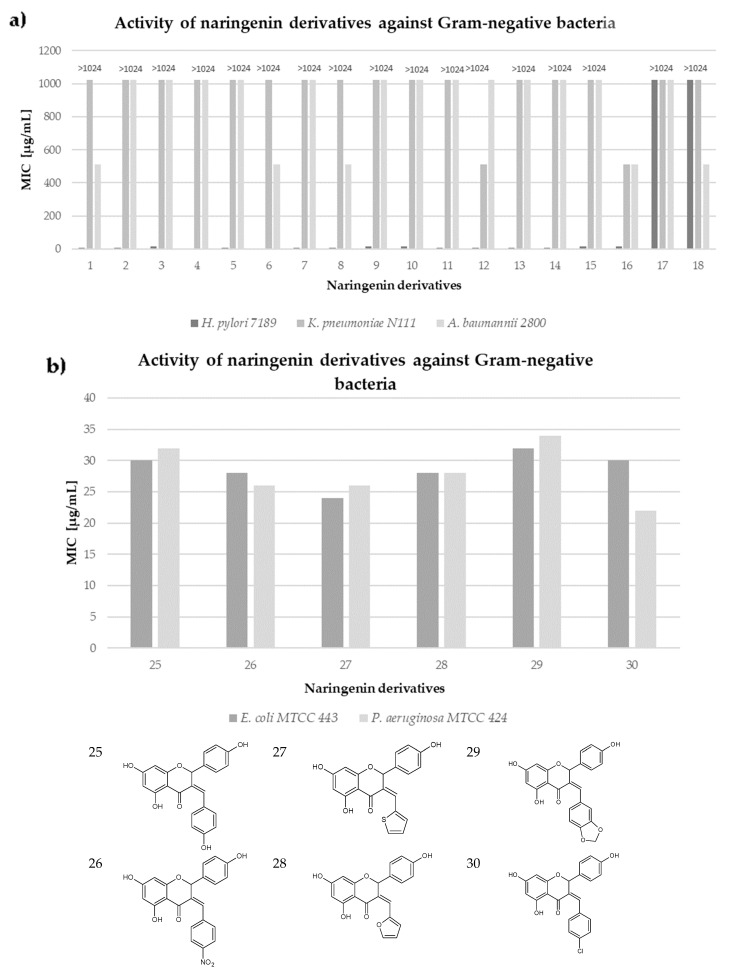
In vitro activity of naringenin derivatives against Gram-negative bacteria. (**a**) 1—7-*O*-methylnaringenin; 2—7-*O*-methylnaringenin oxime; 3—7,4′-di-*O*-methylnaringenin; 4—7,4′-di-*O*-methylnaringenin oxime; 5—7-*O*-ethylnaringenin; 6—7-*O*-ethylnaringenin oxime; 7—7-*O*-isopropylnaringenin; 8—7-*O*-isopropylnaringenin oxime; 9—7,4′-di-*O*-isopropylnaringenin; 10—7,4′-di-*O*-isopropylnaringenin oxime; 11—7-*O*-butylnaringenin; 12—7-*O*-butylnaringenin oxime; 13—7-*O*-pentylnaringenin; 14—7-*O*-pentylnaringenin oxime; 15—7-*O*-hexylnaringenin; 16—7-*O*-hexylnaringenin oxime; 17—7,4′-di-*O*-hexylnaringenin; 18—7,4′-di-*O*-hexylnaringenin oxime (based on Duda-Madej et al. [67]). (**b**) based on Murti et al. [80].

Resistance among Gram-negative bacteria appears to be a bigger problem due to easy spreading by plasmid transfer [76]. The second reason is lack of new antibiotics targeting Gram-negative bacteria or the existing ones being very harmful for the patients. Searching for compounds with promising MIC values is of primary interest to many scientist; often they seek novelties among natural components. The activity of naturally derived compounds against Gram-negative bacteria is usually lower, probably due to the presence of negatively charged lipids in the outer layer of the membrane, which acts like the lipid barrier [77,78]. Despite the fact that naringenin is not a new substance—it was the object of research in the late 80s and early 90s—the compound has been undergoing a renaissance in recent years [79]. Naringenin derivatives have become an object of particular interest. There are many scientific studies describing the activity of plant extracts containing naringenin, but there is not much research on the pure compound, which is the focus of this review. The MIC of naringenin and naringin against Gram-negative bacteria were measured by many authors and are summarized by us in the table below (Table 1). The most frequently tested species were *E. coli*, *H. pylori*, *Pseudomonas aeruginosa* and *K. pneumoniae*. Authors determined the antibacterial activity against different strains of these bacteria, which were either purchased or isolated from patients. The MIC values of naringenin ranged from 0.5–1 up to 2000 µg/mL; however usually 1000 µg/mL was the highest concentration tested. There were similarly good results for *H. pylori,* ranging from 40 µg/mL to 100 µg/mL. The values for *E. coli* heavily differed (12.5–1000 µg/mL). Results of naringin were significantly worse comparing to naringenin. Some close derivatives of naringenin tested by Murti et al. and Duda-Madej et al. had surprisingly satisfying values (MIC ≤ 50 µg/mL) shown in the Scheme 2a,b [67,80]. Beside in vitro measurements of MICs, there are a number of molecular and biochemical studies demonstrating the effect of this bioflavonoid on the Gram-negative bacterial cell. Indeed, it has been shown that naringenin could affect the expression of genes involved in QS (e.g., *lasI, lasR, rhlI, rhlR, lasA, lasB, phzA1* and *rhlA)*, thus reducing the production of pyocyanin and elastin of *P. aeruginosa* PAO1 [81]. It is also known that naringenin exhibits 34% inhibition on the urease of *H. pylori,* although in concentration of 300 µg/mL, which is 3–7.5 times higher than its MIC against *H. pylori* strains [82].

Vikram and co-workers suggested that naringenin attenuates virulence and mobility of *Salmonella enterica* subsp. *enterica* serovar Typhimurium owing to repressing 24 genes of pathogenicity island 1 and down-regulating 17 genes of mobility [83]. The same research team, in another of their studies, demonstrated the inhibitory effect of naringenin on biofilm formation of *Vibrio harveyi* and *E. coli* O157:H7 strains. The bioflavonoid directly disrupted the type III secretion system (T3SSs) of these strains [84]. Other researchers have shown a direct effect of naringenin on the bacterial cell. In the presence of this compound some spectacular changes occurred in the composition of the outer cell membrane of *E. coli* strains. This was due to influence on the expression of fatty acid biosynthesis-associated genes, including *fabG, fabI, fabD*, *cfa* (decrease), and *fabA* (up-regulates) [65]. Although naringenin appears to be a promising substance in the fight against Gram-negative bacteria, similar to current antibiotics it can be neutralized by microbes. *Herbaspirillum seropedicae* has the ability to degrade naringenin, probably owing to SmR1 operon, whose expression is induced by naringenin. Mutation of *fdeA* gene, belonging to SmR1 operon, results in a decline of the ability to degrade naringenin [85]. Complementing the effective fight against Gram-negative bacteria is the search for different forms of naringenin delivery into the body. It has been shown that only 15% of this bioflavonoid is absorbed after oral administration, so treatment by this route is not very promising, and new solutions are required [8,73].

**Table 1:** In vitro activity of naringenin and naringin against Gram-negative bacteria.

Strain	Activity of Naringenin	Reference
*P. aeruginosa* c.i.	128 µg/mL	[86]
*E. coli* ATCC 8739	1000 µg/mL	[87]
*E. coli* ATCC 11775	1000 µg/mL	
*H. pylori* ATCC43504	80 µg/mL	[82]
*H. pylori* NCTC11637	40 µg/mL	
*H. pylori* NCTC11638	40 µg/mL	
*H. pylori* 82516 c.i.g	40 µg/mL	
*H. pylori* 82548 c.i.g	40 µg/mL	
*H. pylori* 4 c.i.g	100 µg/mL	
*E. coli* ATCC 25922	1000 µg/mL	[65]
*E. cloacae* DMST 21394	>512 µg/mL	[88]
*E. cloacae* DMST 21549	>512 µg/mL	
*E. cloacae* DMST 19719	>512 µg/mL	
*E. coli* ATCC 25922	>512 µg/mL	
*K. pneumoniae* ATCC 13883	0.5–1 µg/mL	[89]
*H. pylori* ATCC 43504	100 µg/mL	[90]
*H. pylori* ATCC 51932	100 µg/mL	
*H. pylori* OX.22 c.i.	100 µg/mL	
*H. pylori* OX. 63 c.i.	100 µg/mL	
*H. pylori* OX.64 c.i.	100 µg/mL	
*H. pylori* OX.67 c.i.	100 µg/mL	
*H. pylori* OX.83 c.i.	100 µg/mL	
*Bacteroides galacturonicus* DSM 3978	250 µg/mL	[91]
*Escherichia coli* DSM 1116	>250 µg/mL	
*H. pylori* ATCC 43 504	100 µg/mL	[92]
*E. coli* ATCC 31030	550 µg/mL	[79]
*P. mirabilis* ATCC 25933	550 µg/mL	
*P. aeruginosa* ATCC 10145	500 µg/mL	
*S. enterica subsp. enterica* serovar Paratyphi B(S-3)	600 µg/mL	
*S. enterica* subsp. *enterica* serovar Typhi D(S-58)	400 µg/mL	
*S. enterica* subsp. *enterica* serovar Typhimurium ATCC 14028	600 µg/mL	
*S. sonnei* D1, L1, S2 ATCC 9290	450 µg/mL	
*S. marcescens* ATCC 11105	500 µg/mL	
*S. boydii* C2, L1, S2 ATCC 8700	100 µg/mL	
*S. marcescens* ATCC 27117	400 µg/mL	[93]
*E. coli* ATCC 25922	>2000 µg/mL	[94]
*K. pneumoniae* ATCC 13883	2000 µg/mL	
*P. mirabilis* ATCC 43071	2000 µg/mL	
*P. aeruginosa* ATCC 27857	>2000 µg/mL	
*S. enterica* subsp. *enterica* serovar Typhimurium ATCC 14028	>2000 µg/mL	
*E. coli* MTCC 1652	12.5 µg/mL	[95]
*P. aeruginosa* MTCC 424	23.5 µg/mL	
*E. coli* K-12 MG1655	800 µg/mL	[96]
*S. enterica* subsp. *enterica* serovar Typhimurium LT2	1000 µg/mL	
*P. putida* ATCC 795	1000 µg/mL	
*S. enterica* subsp. *enterica* serovar Typhimurium ATCC 14028	250 µg/mL	[97]
*E. coli* 916 c.i.g	125 µg/mL	[98]
*S. enterica* subsp. *enterica* serovar Typhimurium 450 c.i.g	125 µg/mL	
*E. coli* ATCC 25922	400 µg/mL	[99]
**Strain**	**Activity of Naringin**	**Reference**
*P. aeruginosa* ATCC 9027	1000 µg/mL	[87]
*H. pylori* ATCC43504	>100 µg/mL	[82]
*H. pylori* NCTC11637	>100 µg/mL	
*H. pylori* NCTC11638	>100 µg/mL	
*H. pylori 82516* c.i.g	>100 µg/mL	
*H. pylori 82548* c.i.g	>100 µg/mL	
*H. pylori 4* c.i.g	>100 µg/mL	
*H. pylori* ATCC 43 504	>100 µg/mL	[92]
*E. coli* c.i.	500 µg/mL	[100]
*P. aeruginosa* c.i.	500 µg/mL	
*E. coli* ATCC 31030	900 µg/mL	[93]
*P. mirabilis* ATCC 25933	700 µg/mL	
*P. aeruginosa* ATCC 10145	600 µg/mL	
*S. enterica* subsp. *enterica* serovar Typhimurium ATCC 14028	800 µg/mL	
*S. marcescens* ATCC 27117	600 µg/mL	
*E. coli* ATCC 25922	>2000 µg/mL	[101]
*E. coli* K-12 MG1655	>1000 µg/mL	[96]
*S. enterica* subsp. *enterica* serovar Typhimurium LT2	>1000 µg/mL	
*P. putida* ATCC 795	>1000 µg/mL	

Legend: c.i.—clinical isolates; c.i.g—clinical isolates from gastroscopic samples; OX—clinical isolates obtained from the Microbiology Lab at OUCRU (Oxford University Clinical Research Unit, Ho Chi Minh City, Vietnam) from patients with duodenal and stomach ulcers.

## 7. Antifungal Activity

According to the CDC, in 2021 in the United Stated there were about 7000 deaths from fungal diseases (*CDC*, n.d.). However, this is only an estimated number as many infections were undiagnosed. Fungal diseases may be really serious and life-threatening, especially in immunosuppressed patients, because they usually suffer from invasive fungal infections (IFIs).

The burden of fungal diseases is the trigger point for searching for new antifungal agents. Many studies are focused on naringenin and its derivatives, as among many properties, they exhibit antifungal effects. In the table below, there are gathered MICs of naringenin and its derivatives that reflect their activity against selected fungi (Table 2).

Soberón and co-workers pointed out that *Candida albicans* is a pathogen that frequently causes hospital infections and may be resistant to many antifungal agents. Inspired by Argentinean folk medicine, the authors obtained naringenin from *Tessaria dodoneifolia* ethanolic extract and examined its antifungal activity on two *C. albicans* strains: ATCC 10231 (sensitive to fluconazole) and 12–99 (resistant to fluconazole). Naringenin was active against both tested strains, and—the main conclusion of the experiment—the results obtained suggested that the combination of this bioflavonoid with fluconazole showed a synergistic effect against resistant strains. Naringenin’s MIC value is marked as 40 μg/mL against both *C. albicans* ATCC 10231 and 12–99 (Table 2) [102]. However, when naringenin was used in concentration 83 μg/mL, it had no antifungal activity on *C. albicans* ATCC 10231 or on *Candida* spp. [103].

Moreover, according to Rauha et al., naringenin fails to inhibit growth of *C. albicans* ATCC 10231, *Aspergillus niger* ATCC 16404 and *Saccharomyces cerevisiae* FOMK [84]. Also, naringin showed no antimicrobial activity against these fungi with its MIC value of 1250 μg/mL against *C. albicans* from clinical isolates (Table 2) [101].

Another group of compounds which possess antifungal properties are ether derivatives of naringenin. *O*-Alkyl derivatives of naringenin and their oximes were tested for activity against bacteria and fungi. It turns out that the strongest effect against *Fusarium linii* KB-F1 showed not only naringenin, but also 7-*O*-dodecylnaringenin oxime, 7,4′-di-*O*-dodecylnaringenin and its oxime. These compounds caused complete growth inhibition of this fungal strain. The same effect was observed for naringenin oxime and 7,4′-di-*O*-pentylnaringenin against *A. niger* DSM1957. Based on these results, elongation of the *O*-alkyl chains attached at positions C-7 and C-4′ in naringenin increased the antimicrobial activity. In the case of *C. albicans* DSM1386, oximes of *O*-alkyl derivatives exhibited a stronger effect than just *O*-alkyl derivatives. This is an important observation, which suggest that introducing the oxime group amplified biological properties [30]. The enhancing of the antifungal properties of naringenin is caused also by introducing heterocyclic nuclei. In the study of Murti Y., twelve naringenin derivatives, substituted at C-3 position, were tested for activity against two fungal strains: *A. niger* MTCC 9687 and *C. albicans* MTCC 183. All of these compounds had an antifungal effect, but the 4′-chlorophenyl-substituted naringenin molecule at the C-3 position had the strongest antifungal activity, and its MIC value was 16 μg/mL against both fungal strains (Table 2). Although all of the derivatives showed antifungal activity, there are also moieties that may reduce it. The methoxy-substituted phenyl ring decreased the antifungal effect of naringenin [80]. According to the above-mentioned studies, different naringenin derivatives also have significant biological properties, some of them even greater than naringenin. This knowledge can open up new possibilities in searching for new antifungal agents.

Flavonoids are also examined for their activity against major rice pathogens. The main cause of much plantation damage and food losses are fungal diseases—the most destructive fungus species appears to be *Magnaporthe oryzae (Pyricularia oryzae)*. Rice is a staple food for over 50% of the world’s population, so such crop damage may lead to tragic consequences [104]. The influence of naringenin on this strain was associated with an inhibitory effect on the spore germination with as low as 7 μg of this bioflavonoid [105]. What is more, rice plants actually use naringenin to produce sakuranetin, which have better antifungal properties. On the other hand, *M. oryzae* metabolizes sakuranetin into naringenin, so it lowers the resistance of rice plants [106]. Being aware of this process may be a starting point for further examination of activity against rice pathogens of naringenin.

**Table 2:** In vitro activity of naringenin and its derivatives against selected fungi strains.

Compound	Strain	Activity	Reference
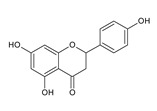	*Candida albicans* ATCC 90028	5 μM	[107]
	*Candida parapsilosis* ATCC 22019	5 μM	
	*Trichosporon beigelii* KCTC 7707	2,5 μM	
	*Malassezia furfur* KCTC 7743	10 μM	
	*Trichophyton rubrum* KCTC 6345	5 μM	
	*Aspergillus flavus* KCTC 6905	10 μM	
	*Saccharomyces cerevisiae* KCTC 7296	5 μM	
	*Candida albicans* ATCC 10231	40 μg/mL	[102]
	*Candida albicans* 19–22	40 μg/mL	
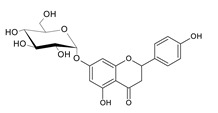	*Candida albicans* ATCC 10231	16 mg/mL	[108]
	*Candida kruzei* ATCC 6258	32 mg/mL	
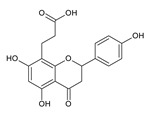	*Aspergillus niger* 439	100 μg/mL	[109]
	*Fusarium oxysporum* (M42)	n.i.e.	
	*Candida albicans* (N/A)	n.i.e.	
	*Saccharomyces cerevisiae* (N/A)	n.i.e.	
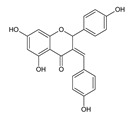	*Candida albicans* MTCC 183	22 μg/mL	[80]
	*Aspergillus niger* MTCC9687	20 μg/mL	
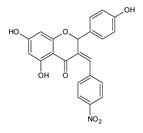	*Candida albicans* MTCC 183	18 μg/mL	[80]
	*Aspergillus niger* MTCC9687	18 μg/mL	
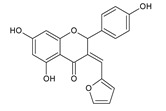	*Candida albicans* MTCC 183	24 μg/mL	[80]
	*Aspergillus niger* MTCC9687	20 μg/mL	
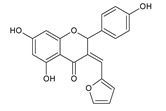	*Candida albicans* MTCC 183	22 μg/mL	[80]
	*Aspergillus niger* MTCC9687	22 μg/mL	
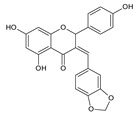	*Candida albicans* MTCC 183	22 μg/mL	[80]
	*Aspergillus niger* MTCC9687	24 μg/mL	
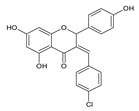	*Candida albicans* MTCC 183	16 μg/mL	[80]
	*Aspergillus niger* MTCC9687	16 μg/mL	

Legend: N/A—not available; n.i.e.—no inhibitory effect.

## 8. The Mechanism of Action of Naringenin on Bacteria and Fungi

Naringenin, like all flavonoids, blocks the respiratory chain, fatty acid synthesis, gyrase activity, QS molecules and increases membrane permeability [110].

Song and co-workers showed in their research that the presence of naringenin not only influences the distribution of fatty acids in the bacterial cell, but also reduces the expression of genes involved in biofilm formation. This situation contributes to a change in the structure of the biofilm and an increase in the sensitivity of the *S. aureus* strain showing MR resistance to antibiotics [70]. Other studies have also shown that naringenin is an antagonist of cell-to-cell signaling, an important regulatory factor in biofilm production in some bacteria [84]. The results obtained by Paczkowski et al. provide additional facts in this regard. They showed that this bioflavonoid has the ability to simultaneously inhibit the pairs LasI/R and RhlI/R, i.e., two synthetases with their receptors, which are responsible for the synthesis of two autoinducers in QS in *P. aeruginosa* [111].

It is significant that flavonoids, including the naringenin discussed in this review, have been shown to use different antimicrobial mechanisms than the antibiotics available on the market [112]. Thus, they can play a huge role in amplifying the synergism of antimicrobial therapy. The available literature data clearly indicate that naringenin interacting with antibiotics enhanced both the synergistic and additive effect. Hyperadditional synergism has been demonstrated in the interaction of this flavonoid with oxacillin, a β-lactam antibiotic [70]. An additive and synergistic effect was also observed in the combination of *O*-alkyl naringenin derivatives with antibiotics from the groups macrolides, fluoroquinolones, nitroimidazoles, aminoglycosides, glycopeptides and polypeptides. Duda-Madej and co-workers speculate that this phenomenon occurs most likely because naringenin and its derivatives must act on the same target sites as the antibiotics used, namely the cell wall, genetic material and/or protein synthesis [67]. Given the fact of the confirmed synergistic effect of naringenin in combination with antibiotics and the ability of this bioflavonoid to increase the activity of liver enzymes (ALT—alanine aminotransaminase; AST—aspartate aminotransferase) [36,113], due caution should be exercised during antibiotic therapy, remembering that these enzymes are responsible for metabolizing drugs. Therefore, the administration of this bioflavonoid at the same time as an antibiotic may contribute to a significant decrease or a dangerous increase (depending on the antibiotic used) in its concentration in the body. Ultimately, this can result in the failure of the antimicrobial therapy or the occurrence of various side effects.

Tsuchiya et al., conducting research on the MRSA strain, proved that the antimicrobial effect of naringenin consists of reduced fluidity in the hydrophilic and hydrophobic regions of both the inner and outer cell membranes [114]. The bacterial plasma membrane is responsible for the processes of self-regulation, respiration, transport, biosynthesis and cross-linking of peptidoglycan, as well as lipid biosynthesis. Thus, disruption of its integrity may directly or indirectly affect metabolic processes and lead to the death of the bacterial cell. Moreover, the ability of the bioflavonoid to block the synthesis of the cell envelope by inhibiting the effect on 3-hydroxylacyl-ACP dehydratase in *H. pylori* and 3-ketoacyl-ACP synthase in *E. faecalis* has been proven [115,116]. These enzymes are necessary for the synthesis of, inter alia, lipoproteins, phospholipids and lipopolysaccharides, i.e., components of the cell membrane. The inhibitory effect on these pathways disrupts the proper functioning of the bacterial cell and represents the hook for disrupting their proliferation. This ability of naringenin is very important as it is desirable in the development of new antibacterial drugs.

Studies on the antimicrobial mechanism of naringenin indicate its active participation in the inhibition of the efflux pumps, i.e., proteins involved in pumping out the antibiotic from the bacterial cell [117]. This bioflavonoid ability has the potential to be developed in the future as a phytobiotic in the fight against microbial resistance. Additionally, the studies by Oh et al. confirm this fact, as they have shown that phenolic compounds (including naringenin) reduce the expression level of CmeABC, a multi-drug efflux pump that is important in antibiotic resistance in *Campylobacter jejuni* strains [118].

## 9. Conclusions

Naringenin, commonly found in large amounts in citrus fruits, is known for many biological activities, e.g., the antioxidant, anti-inflammatory, anti-cancer, but also antimicrobial. Naringenin itself is characterized by a much lower activity against Gram-negative bacteria, in contrast to Gram-positive bacteria, where this activity is much more satisfactory. In turn, its antifungal activity is at a moderate level against both filamentous and sporulation fungi. Very promising results in antimicrobial activity are given by naringenin derivatives, which emphatically improve the antimicrobial activity. The incorporation of the oxime group in place of the carbonyl moiety at the C-4 position significantly increases the activity against pathogenic microorganisms, both Gram-positive (e.g., *S. aureus*, *E. faecalis*) and Gram-negative (e.g., *H. pylori*, *E. coli*, *P. aeruginosa*). A similar relationship was observed in studies on antifungal activity. Pure naringenin showed even more than two times lower activity than its derivatives (containing, among others: *O*-alkyl chains; 4′-hydroxyphenyl, 4′-nitrophenyl, 4′-chlorophenyl, thiophene, furfural or piperonal rings). The comprehensive action of naringenin, which we focus on in this review, suggests the need for further research on this bioflavonoid and its derivatives and their relationship with pathogenic microorganisms and, consequently, their effects on the human body.

## Data Availability

Not applicable.
